# GIRK Channels as Candidate Targets for the Treatment of Substance Use Disorders

**DOI:** 10.3390/biomedicines10102552

**Published:** 2022-10-13

**Authors:** Hiroko Kotajima-Murakami, Soichiro Ide, Kazutaka Ikeda

**Affiliations:** Addictive Substance Project, Tokyo Metropolitan Institute of Medical Science, 2-1-6 Kamikitazawa, Setagaya-ku, Tokyo 156-8506, Japan

**Keywords:** GIRK channels, substance use disorder, ifenprodil, alcohol, methamphetamine, clinical trials

## Abstract

Substance use disorders (SUDs) are chronic, lifelong disorders that have serious consequences. Repeated substance use alters brain function. G-protein-activated inwardly rectifying potassium (GIRK) channels are expressed widely in the brain, including the reward system, and regulate neuronal excitability. Functional GIRK channels are identified as heterotetramers of GIRK subunits (GIRK1–4). The GIRK1, GIRK2, and GIRK3 subunits are mainly expressed in rodent brain regions, and various addictive substances act on the brain through GIRK channels. Studies with animals (knockout and missense mutation animals) and humans have demonstrated the involvement of GIRK channels in the effects of addictive substances. Additionally, GIRK channel blockers affect behavioral responses to addictive substances. Thus, GIRK channels play a key role in SUDs, and GIRK channel modulators may be candidate medications. Ifenprodil is a GIRK channel blocker that does not have serious side effects. Two clinical trials were conducted to investigate the effects of ifenprodil in patients with alcohol or methamphetamine use disorder. Although the number of participants was relatively low, evidence of its safety and efficacy was found. The present review discusses the potential of GIRK channel modulators as possible medications for addiction. Therapeutic agents that target GIRK channels may be promising for the treatment of SUDs.

## 1. Introduction

Substance use disorders (SUDs) are characterized by compulsive drug use despite clinically significant distress and other negative consequences in life. Substance use disorders alter how the brain and body respond to addictive substances, and patients with SUDs suffer from an inability to quit addictive substances. Addictive substances comprise natural, semi-synthetic, and synthetic substances, such as amphetamine/methamphetamine, cocaine, opioids, cannabinoids, alcohol, hypnotics/anxiolytics, inhalants, nicotine, and caffeine. Addictive-substance misuse and problems that are associated with SUDs have been a serious societal concern worldwide. In 2022, the United Nations Office on Drugs and Crime reported on opioid-related overdose deaths in North America and Canada, the distribution of quantities of heroin and morphine seized in the Balkan route (the Islamic Republic of Iran, half of Transcaucasia, and South Eastern Europe), expansion of methamphetamine production in Afghanistan, distribution of quantities of cannabis resin seized, and trafficking in North Africa, Western and Central Europe, and South-West Asia [1]. However, research on pharmacotherapies for SUDs is still in an incipient stage. To develop new therapeutic agents, we need to focus on the mechanisms that contribute to SUDs. The present review focuses on G-protein-activated inwardly rectifying potassium (GIRK) channels. GIRK channels are expressed in the central nervous system, including brain regions that are related to reward and regulate neuronal excitability. Here, we highlight the involvement of GIRK channels in SUDs and describe the relevance of GIRK channels as potential treatment targets for patients with SUDs.

## 2. Fundamental Function of GIRK Channels and Response to Addictive Substances

**Figure 1:**
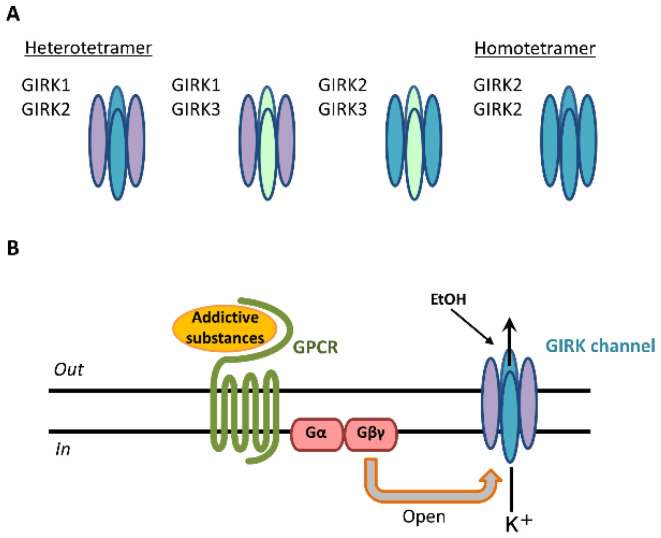
GIRK channel subunits in the brain and signal transduction of addictive substances through GIRK channels. Three types of GIRK subunits are expressed and form heterotetramers (GIRK1/GIRK2, GIRK1/GIRK3, GIRK2/GIRK3) and homotetramers (GIRK2/GIRK2) in various brain regions (**A**). Addictive substances or neurotransmitters bind to GPCRs, and the GIRK channel is activated by the G protein G_βγ_ subunit. Following this activation, potassium ions (K^+^) flow into the cell, and cellular excitability decreases (**B**).

GIRK channels are encoded by the *Kcnj* gene and assembled in homotetrameric or heterotetrameric units comprising four subunits: GIRK1 (Kir3.1/Kcnj3), GIRK2 (Kir3.2/Kcnj6), GIRK3 (Kir3.3/Kcnj9), and GIRK4 (Kir3.4/Kcnj5) [2,3,4]. Each GIRK subunit possesses two transmembrane domains, TM1 and TM2, that flank a hydrophobic pore domain and intracellular N- and C-terminal domains [4]. GIRK1-3-containing channels are widely expressed in the rodent brain, including the cerebral cortex, amygdala, hippocampus, thalamus, ventral tegmental area (VTA), locus coeruleus, and cerebellum. GIRK channels are expressed in the brain’s reward system [5,6]. The expression of GIRK4 subunits is found mainly in the heart and limited to only a few brain regions, such as the neocortex, insular cortex, cerebellar cortex, hypothalamus, thalamus, and brainstem [7,8]. Three primary GIRK channel subunits in the brain form heteromeric channels: GIRK1/GIRK2, GIRK1/GIRK3, and GIRK2/GIRK3 [4] (Figure 1A).

GIRK2 subunits can uniquely form functional homomers in the brain [4]. These various compositions exhibit K^+^ selectivity, inward rectification, and G-protein-dependent gating [2,4]. Outward K^+^ currents through GIRK channels inhibit cellular excitability [2,4]. The core reward circuitry in the brain consists of the VTA, nucleus accumbens (NAc), and ventral pallidum via the medial forebrain bundle. The VTA is the initiating nucleus of the dopaminergic system, which then projects to the NAc via the medial forebrain bundle [9]. Dopamine neurons in the VTA also project to the amygdala, orbitofrontal cortex, anterior cingulate cortex, hippocampus, and prefrontal cortex [10]. Dopamine neurons in the VTA express only the GIRK2 and GIRK3 subunits, whereas γ-aminobutyric acid (GABA) neurons in the VTA express the GIRK1, GIRK2, and GIRK3 subunits [5,6]. Addictive substances enhance reward circuitry in the brain and produce feelings of pleasure. These “rewarding effects” positively reinforce their use and increase relapse risk. Addictive substances include alcohol, nicotine, caffeine, psychostimulants (amphetamines, methamphetamines), cocaine, and opioids [11]. Activation by these substances is mediated by interactions with G-protein-coupled receptors (GPCRs), including GABA receptors, *N*-methyl-D-aspartate (NMDA) receptors, nicotinic acetylcholine receptors, adenosine receptors, and opioid receptors, as well as dopamine transporters [10,12,13,14,15,16,17,18,19]. Following the stimulation of GPCRs, GIRK channels are activated through coupling of the G_i/o_ family of G-proteins to those receptors and gated by G-protein βγ subunits that are released from the G-protein α subunit [20,21] (Figure 1B). G-protein βγ subunits activate GIRK channels through direct binding with amino- and carboxyl-ends of the channels [2,22]. The G-protein α subunit was not thought to be responsible for GIRK channel activation, but rather to be a regulator of the specificity of channel activation [4,23]. Among addictive substances, alcohol activates GIRK channels in a G-protein-independent manner [24], whereas cocaine and phencyclidine directly inhibit GIRK channels at toxic concentrations [25,26]. Early studies in mice that lacked GIRK channel subunits suggested that GIRK channels play an important role in regulating behavioral effects of addictive substances (Table 1).

GIRK1 knockout and GIRK2 knockout mice exhibited baseline hyperactivity and an increase in locomotor response to cocaine [27]. GIRK1 knockout and GIRK2 knockout mice exhibited a decrease in analgesic responses after morphine administration into the spinal cord [28]. GIRK2 knockout and GIRK3 knockout mice also exhibited a decrease in cocaine self-administration [30]. GIRK2 knockout mice did not exhibit alcohol-induced conditioned taste aversion or conditioned place preference [31]. Morphine-induced activity increased in GIRK1 knockout and GIRK2 knockout mice but decreased in GIRK3 knockout mice compared with wildtype mice [29]. GIRK3 knockout mice exhibited a reduction of alcohol withdrawal [7] and an alcohol-induced conditioned place preference [37]. Additionally, GIRK3 knockout mice exhibited a selective increase in alcohol binge-like drinking without affecting alcohol metabolism or the sensitivity to alcohol intoxication [38]. The roles of GIRK channels have also been studied using *weaver* mutant mice, which have spontaneously occurring autosomal recessive mutations of the *Girk2* gene that lead to a reduction of GIRK2 channel function and cause abnormalities of dopamine signaling [39]. *Weaver* mutant mice exhibited lower antinociceptive effects of alcohol [24] and opioids [32]. Amphetamine caused less hyperlocomotion in *weaver* mutant mice [33]. *Weaver* mutant mice also did not exhibit methamphetamine-induced conditioned place preference or priming effects [34]. A significant decrease in basal and methamphetamine-induced dopamine release was also detected in the NAc, with a decrease in methamphetamine-induced neural activity in the posterior NAc shell [34]. Furthermore, neuron-specific knockout mice have been generated to investigate the role of GIRK channels in the reward system. McCall et al. (2017) reported that the genetic ablation of GIRK2 in dopamine neurons, which did not alter the baseline excitability of VTA dopamine neurons, increased behavioral sensitivity to cocaine [35]. The overexpression of GIRK3 in VTA dopamine neurons decreased GABA_B_ receptor- and dopamine D_2_ receptor-dependent signaling and increased cocaine-induced locomotion, whereas the overexpression of GIRK2 increased GABA_B_ receptor-dependent signaling and decreased cocaine-induced locomotion [36]. Knockout and missense mutation mice are useful for studying the response of GIRK channels to addictive substances, but the response to addictive substances may differ between mice with knockouts and missense mutations. This may be attributable to the complete or functional deficiency of GIRK channels. The relevance of GIRK channel function in VTA dopamine neurons has been demonstrated, and GIRK channels have been shown to play a key role in behavioral responses to addictive substances. In human studies, Nishizawa et al. reported associations between GIRK channels and addictive substances by analyzing single-nucleotide polymorphisms (SNPs). Human gene polymorphism analysis revealed that the rs2835859 SNP in the *KCNJ6* gene, which encodes the GIRK2 subunit, was associated with nicotine dependence [40]. Single-nucleotide polymorphisms at A1032G in the *KCNJ6* gene were reported to be associated with sensitivity to opioids after major abdominal surgery [41]. These previous animal and human studies indicate that GIRK channels shape many behavioral responses to addictive substances, suggesting that medications that target GIRK channels may be useful for treating patients with SUDs.

## 3. Pharmacological Modulation of GIRK Channels and Therapeutic Effects

Although GIRK channels are activated in a G-protein-dependent or -independent manner by addictive substances, antipsychotic compounds, psychoactive compounds (e.g., antidepressants, including selective serotonin re-uptake inhibitors), and a cerebral circulation/metabolism ameliorator were shown to block brain-type GIRK1/GIRK2 channels and cardiac-type GIRK1/GIRK4 channels in the *Xenopus* oocyte expression assay [42,43,44,45,46,47]. Interestingly, fluoxetine, desipramine, paroxetine, sertraline, and ifenprodil blocked alcohol-induced GIRK1/GIRK2 currents [43,44,45,46,47]. Ifenprodil can also inhibit GIRK channels at a lower IC_50_ than antidepressants (e.g., fluoxetine, imipramine, desipramine, amitriptyline, nortriptyline, clomipramine, maprotiline, paroxetine, sertraline, duloxetine, and amoxapine) [43,44,45,46,47]. Each GIRK channel was inhibited by ifenprodil at the following IC_50_ values: 7.01 ± 0.92 μM at GIRK1/GIRK2, 8.76 ± 1.26 μM at GIRK2, and 2.83 ± 0.69 μM at GIRK1/GIRK4 [46]. Although these compounds do not have specificity for GIRK channels, some of these compounds were shown to inhibit behaviors that are induced by addictive substances. Takamatsu et al. reported that pretreatment with fluoxetine and paroxetine inhibited methamphetamine-induced conditioned place preference in mice, which was unaffected by fluvoxamine, an antidepressant that does not block GIRK channels [48,49]. However, paroxetine has several adverse effects, including serotonin syndrome, neuroleptic malignant syndrome, convulsions, toxic epidermal necrosis, antidiuretic hormone incompatible secretion syndrome, severe liver dysfunction, rhabdomyolysis, low white blood cell counts, and anaphylaxis [50]. The U.S. Food and Drug Administration (FDA) approved fluoxetine, but it is not currently approved for use in Japan. Ifenprodil is a blocker of α_1_-adrenergic receptors, GluN2B subunit-containing NMDA receptors [51,52], and sigma-1/2 receptors [53], and also inhibits GIRK channels [46,51]. Ifenprodil inhibited methamphetamine-induced conditioned place preference [54], and pretreatment with ifenprodil reduced morphine-induced conditioned place preference in mice [55]. Ifenprodil also inhibited the amphetamine-induced potentiation of excitatory postsynaptic currents in rat midbrain dopamine neurons [56]. Pretreatment with a combination of ifenprodil and cyproheptadine in mice did not cause locomotor sensitization compared with mice that were pretreated with saline when the mice were repeatedly injected with D-amphetamine [57]. Notably, ifenprodil inhibits GluN2B subunit-containing NMDA receptors at a lower IC_50_ than GIRK channels (IC_50_: 0.34 μM at GluN1A/GluN2B receptors; see IC_50_ values for GIRK channels above) [51]. A recent study reported that the methamphetamine-induced increase in locomotor activity (i.e., behavioral sensitization) was blocked by ifenprodil via GluN2B-protein phosphatase 2A-AKT signaling in the dorsal striatum in mice [58]. These studies suggest that ifenprodil inhibits behaviors that are induced by addictive substances through GluN2B subunit-containing NMDA receptors. Thus, ifenprodil may exert effects on behaviors that are caused by addictive substances through NMDA receptors and/or GIRK channels. Kotechi et al. reported that GIRK2/GIRK3 channels in VTA dopamine neurons regulated morphine-induced motor activity, whereas GIRK channel activation in VTA GABA neurons was not required [29]. GIRK channels play a key role in the influence of addictive substances on the reward system, but further studies are warranted to define the mechanisms of action of ifenprodil.

Ifenprodil has been used as a treatment for dizziness after brain ischemia (<60 mg/day) in a few countries, including Japan and France. A high dose of ifenprodil (60–300 mg/kg, which is not covered by insurance in Japan) is used as an analgesic in patients with cancer in Japan [59]. Ifenprodil does not have serious adverse effects [60]. Table 2 shows clinical studies of GIRK channel inhibitors, including ifenprodil, in SUDs.

**Figure 2:**
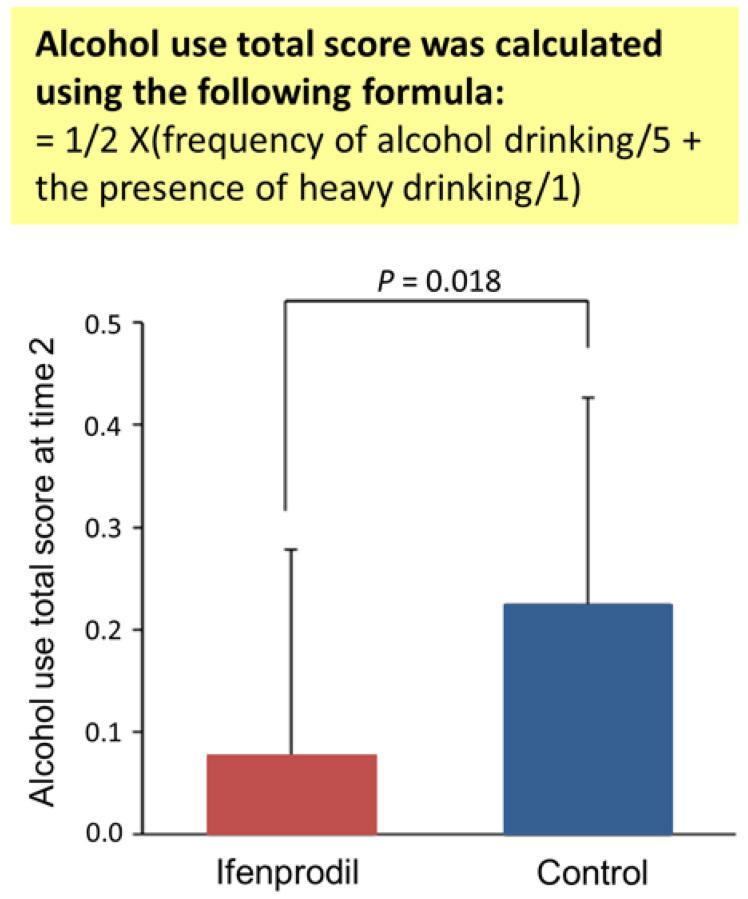
Effects of ifenprodil treatment on alcohol use scores. Weighted mean of the frequency of alcohol drinking and the presence or absence of heavy drinking were calculated as the alcohol use total score. The frequency of alcohol drinking was assessed by asking the following question: “How often did you use alcohol for the past month?” Respondents answered by selecting one of six choices (0 = none, 1 = once per month, 2 = two to four times per month, 3 = two or three times per week, 4 = four to six times per week, and 5 = every day). The presence or absence of heavy drinking was assessed by asking the following question: “Have you drunk heavily for the past month?” Respondents answered “Yes” or “No” (1 = Yes, 0 = No). The difference in alcohol use scores at time 2 (after 3 months) was calculated after adjusting the score at baseline (time 1). The number of participants was the following: ifenprodil (*n* = 25), control (*n* = 21). The data were analyzed using analysis of covariance. Error bars indicate standard deviations. The figure was modified from Sugaya et al., 2018 [64]. Reprinted/adapted with permission from Ref. [64]. Copyright year; 2018, copyright owner’s name; Kazutaka Ikeda.

Goto reported that ifenprodil diminished pain in the extremities, headache, and tremors in the fingers in patients with alcohol use disorder [61]. In a retrospective chart review of patients with alcohol use disorder, patients who took GIRK channel inhibitors, including ifenprodil, were compared to patients who did not take GIRK channel inhibitors [62,63]. These studies showed that GIRK channel inhibition improved the lack of negative expectancy for drinking and the positive expectancy for alcohol, which are components of relapse risk on the Stimulant Relapse Risk Scale (SRRS), in outpatients and inpatients [62,63]. These results suggest that ifenprodil may benefit from a redesign as a therapeutic agent for patients with SUDs. We conducted two clinical trials to investigate the effects of ifenprodil in patients with SUDs in Japan. In the first clinical trial, patients with alcohol use disorder were examined. The study had a randomized, controlled, rater-blinded, single-center design, and the drug administration period was 3 months [64]. This study found that ifenprodil (60 mg/day) administration for 3 months improved alcohol use scores in patients with alcohol use disorder (Figure 2), and no patients reported adverse events from ifenprodil [64].

In the second clinical trial, the effects of ifenprodil were examined in patients with methamphetamine use disorder [65,67]. Methamphetamine is one of the most abused drugs in Japan [68]. This study was conducted in Japan and had a randomized, double-blind, exploratory, dose-ranging, placebo-controlled, single-center design. Three arms (placebo, 60 mg/day ifenprodil, and 120 mg/day ifenprodil groups), including high-dose ifenprodil, were set in the study because 120 mg/day ifenprodil suppressed craving without adverse events in a patient who was addicted to various substances (Bron^®^, cough medicine, and alcohol) [66]. The administration period was 84 days (12 weeks), with the following numbers of participants: placebo group (*n* = 10), 60 mg/day ifenprodil group (*n* = 11), and 120 mg/day ifenprodil group (*n* = 11). The primary outcome was the use or nonuse of methamphetamine during the drug administration period in the placebo group vs. the 120 mg/day ifenprodil group (see our previous studies for secondary outcomes). In this clinical trial, we did not find effects of ifenprodil on the primary or secondary outcomes [65,67]. The additional analyses, however, showed that the number of days of methamphetamine use during the follow-up period was lower, and emotional problems on the SRRS improved after treatment with 120 mg/day ifenprodil compared with both the placebo and 60 mg/day ifenprodil groups [65,67]. Importantly, there were no adverse events associated with ifenprodil or placebo administration. However, these two clinical trials had a common limitation—that is, a relatively low number of patients. Future clinical trials should include larger samples to assess more precisely the efficacy of ifenprodil treatment. Our studies were the first clinical trials on the treatment of SUDs (alcohol and methamphetamine) with ifenprodil, demonstrating the safety of ifenprodil in patients with SUDs. Further clinical studies should evaluate ifenprodil as a pharmacotherapy for SUDs. Recent studies reported a new class of subunit-selective GIRK channel modulators (GIRK1-containing or non-GIRK1-containing GIRK channels) [69]. ML297 (VU0456810) selectively activates GIRK1-containing heteromers and prevents epilepsy [70]. V0529331 is a synthetic small molecule that was reported to activate non-GIRK1-containing GIRK channels. The discovery of this molecule may be useful for developing selective non-GIRK1-containing GIRK channel probes [71]. Crystal structures of the GIRK2 channel and G-protein βγ subunit were analyzed [72]. In 2019, potassium channel tetramerization domain-containing protein, a modified subunit of the GABA_B_ receptor, was shown to modulate the kinetics of GIRK channels, resulting in rapid desensitization [73]. These studies could be useful for identifying the role of GIRK channel subunits and for developing medications that are specific to GIRK2/GIRK3 channels in VTA dopamine neurons, which are at the hub of reward circuitry in the brain.

## 4. Future Directions

Substance use disorders are chronic diseases. Patients with SUDs and associated societal problems exist worldwide, including in Japan [1]. The FDA has approved several medications for the treatment of alcohol and opioid use disorders in combination with counseling and behavioral therapies [74]. Acamprosate, disulfiram, and naltrexone are used for alcohol use disorder. Buprenorphine, methadone, naltrexone, and naloxone are used for opioid use disorder. No medications have yet been approved for methamphetamine use disorder. The development of new medications and availability of more treatment options would undoubtedly be beneficial for patients with SUDs. The present review focused on SUDs, but addictioncan occur beyond SUDs, such as behavioral addictions [75]. Although the objects on which individuals depend are different, the reward system in the brain plays key roles in both SUDs and behavioral addictions [76]. Future studies should test the efficacy of ifenprodil and other medications for the treatment of behavioral addictions.

## 5. Concluding Remarks

GIRK channels are essential mediators of cellular excitability in the central nervous system and are involved in the rewarding effects of addictive substances. Previous studies with GIRK channel knockout and mutant mice confirmed that GIRK channels are involved in regulating behavioral responses to addictive substances. Our two clinical trials suggest that ifenprodil might be effective for patients with alcohol use disorder and methamphetamine use disorder, without causing severe adverse events. Ifenprodil is not a selective GIRK channel blocker; it also inhibits α_1_-adrenergic receptors, GluN2B-containing NMDA receptors, and sigma-1/2 receptors. Future studies should clarify the mechanisms by which ifenprodil acts to treat SUDs. Understanding the molecular and structural bases by which addictive drugs act on GIRK channels and how they change their gating and conformation properties will provide new insights into the development of novel treatments for SUDs.

## Figures and Tables

**Table 1:** GIRK channel genes manipulations and substance-related behaviors.

Gene	Genetic Modification	Drug	Result	Reference
*Girk1*	KO	Cocaine	⬆ Motor activity	Arora et al., 2010 [27]
		Morphine	⬇ Antinociceptive effects	Marker et al., 2004 [28]
		Morphine	⬆ Motor activity	Kozell et al., 2009 [7]
		Morphine	⬆ Motor activity	Kotecki et al., 2015 [29]
*Girk2*	KO	Cocaine	⬇ Self-administration	Morgan et al., 2003 [30]
		Cocaine	⬆ Motor activity	Arora et al., 2010 [27]
		Alcohol	⬇ Conditioned place preference/Conditioned taste aversion	Hill et al., 2003 [31]
		Morphine	⬆ Motor activity	Kotecki et al., 2015 [29]
		Morphine	⬇ Antinociceptive effects	Marker et al., 2004 [28]
	Missense mutation	Alcohol	⬇ Antinociceptive effects	Kobayashi et al., 1999 [24]
		Opioids	⬇ Antinociceptive effects	Ikeda et al., 2000 [32]
		Amphetamine	⬇ Motor activity	Schmidt et al., 1982 [33]
		Methamphetamine	⬇ Conditioned place preference	Ikekubo et al., 2020 [34]
	KO in DA neurons	Cocaine	⬆ Behavioral sensitivity	McCall et al., 2017 [35]
	Overexpression in DA neurons	Cocaine	⬇ Motor activity	McCall et al., 2019 [36]
*Girk3*	KO	Cocaine	⬇ Self-administration	Morgan et al., 2003 [30]
		Morphine	⬇ Motor activity	Kotecki et al., 2015 [29]
		Alcohol	⬇ Withdrawal	Kozell et al., 2009 [7]
		Alcohol	⬆ Conditioned place preference	Tipps et al., 2016 [37]
		Alcohol	⬆ Binge-like drinking	Herman et al., 2015 [38]
	Overexpression in DA neurons	Cocaine	⬆ Motor activity	McCall et al., 2019 [36]

KO: knock out, DA: dopamine. Up arrow: Increased behavioral phenotype, Down arrow: Decreased/inhibited behavioral phenotype.

**Table 2:** Clinical studies of GIRK channel inhibitors in SUDs.

Treatment	Study Design	Drug	Result	Reference
Ifenprodil	Case report *	Alcohol	⬇ Pain in the extremities and headache ⬇ Tremors in the fingers	Goto, 2010 [61]
Ifenprodil, Paroxetine, and Haloperidol	Retrospective chart review **	Alcohol	⬇ The lack of negative expectancy for drinking on the SRRS	Ogai et al., 2011 [62]
Ifenprodil, Paroxetine, and Sertraline	Retrospective chart review **	Alcohol	⬇ The positive expectancy for alcohol on the SRRS	Sugaya et al., 2012 [63]
Ifenprodil	Randomized, controlled, rater-blinded study	Alcohol	⬇ Alcohol use scores	Sugaya et al., 2018 [64]
Ifenprodil	Randomized, double-blind, exploratory, dose-ranging, placebo-controlled study	Methamphetamine	⬇ The days of methamphetamine use during the follow-up period	Kotajima-Murakami et al., 2022 [65]
Ifenprodil	Case report *	Alcohol Bron	⬇ Craving ⬇ Craving	Hori et al., 2010 [66]

*: Abstract of Japanese conference; **: reports in Japanese academic journals; SRRS: Stimulant Relapse Risk Scale. Down arrow: Decreased the effects of addictive substances.

## Data Availability

Not applicable.

## Abbreviations

SUD	Substance use disorder
GIRK	G-protein-activated inwardly rectifying potassium
VTA	Ventral tegmental area
NAc	Nucleus accumbens
GPCR	G-protein-coupled receptor
NMDA	*N*-methyl-D-aspartate
GABA	γ-aminobutyric acid
SNP	Single-nucleotide polymorphism
SRRS	Stimulant Relapse Risk Scale
